# An Evaluation of Three Ways of Communicating Carrier Status Results to the Parents of Children in a Neonatal Sickle Cell Screening Programme

**DOI:** 10.3389/fped.2020.00300

**Published:** 2020-06-19

**Authors:** Christelle Rémus, Aurélie Stanislas, Naïm Bouazza, Valérie Gauthereau, Michel Polak, Stéphane Blanche, Assa Niakaté, Eliane Gluckman, Jean-Marc Tréluyer, Arnold Munnich, Robert Girot, Marina Cavazzana

**Affiliations:** ^1^Département de Génétique, Hôpital Necker-Enfants Malades, Assistance Publique-Hôpitaux de Paris, Paris, France; ^2^Centre d'Investigation Clinique de Biothérapie, Hôpital Necker-Enfants Malades, Assistance Publique-Hôpitaux de Paris, Paris, France; ^3^Département de Biothérapie, Hôpital Necker-Enfants Malades, Assistance Publique-Hôpitaux de Paris, INSERM, Paris, France; ^4^Unité de Recherche Clinique Necker-Cochin, Assistance Publique-Hôpitaux de Paris - EA 7323, Université Paris Descartes Sorbonne Paris Cité, Paris, France; ^5^Fédération Parisienne pour le Dépistage, la Prévention du Handicap chez l'Enfant, Paris, France; ^6^Unité d'Immuno-Hématologie Pédiatrique, Hôpital Necker-Enfants Malades, Assistance Publique-Hôpitaux de Paris, Paris, France; ^7^Centre d'Information et de Dépistage de la Drépanocytose, Paris, France; ^8^Centre Scientifique de Monaco, Eurocord, Hopital Saint Louis, Université Paris Diderot, Paris, France

**Keywords:** sickle cell disease, sickle cell disease trait, parental notification, screening, follow-up, phone call, text messaging, letter

## Abstract

**Aim:** Sickle cell disease (SCD) is the most frequent monogenic disease worldwide; ~5–7% of the world population carry a hemoglobin disorder trait. In the US, one in every 1,941 newborns has SCD, whereas one in every 3,000 newborns in France is affected - resulting in 385 new cases and 5,883 newly identified carriers per year. The objective of the present study was to evaluate three different ways of providing information to parents at risk of having a child with SCD, with a view to increasing the parental screening rate and decreasing the number of new cases per year in France.

**Method:** In a randomized study, we contacted 300 couples of parents after their child had been identified as a SCD carrier in the French national newborn screening programme: 100 couples received an information letter (the standard procedure in France: arm A), 100 couples received a letter and then a follow-up phone call (arm B), and 100 received a letter and then three follow-up text messages at 5-day intervals (arm C). The primary endpoint was the number of parents in each arm screened in the 120 days after the letter had been sent. In a modified intention-to-treat analysis, the screening rate was 17% in arm A, 35% in arm B, and 30% in arm C.

**Results:** Telephone and text message follow-ups were associated with higher screening rates, compared with no follow-up. After being informed of their child's carrier status, some parents had consulted a healthcare professional but had not been referred for screening (16% in arm A, 19% in arm B, and 13% in arm C).

**Conclusion:** A letter followed by a phone call or three text messages is more effective than a letter alone for informing parents at risk of having a child with SCD. The effective implementation of this follow-up programme probably requires better training of all the healthcare professionals involved.

## Introduction

The primary objective of neonatal sickle cell screening is to identify newborns with SCD (1). However, a secondary objective in France is to identify SCD carriers (i.e., HbAS and HbAC carriers) as recommended by the French National Ethical Committee (https://www.ccne-ethique.fr/sites/default/files/publications/avis097.pdf). Given that HbAS/HbAC carriers have the same genetic risk of having a child with a major sickle cell syndrome, we considered both in the present study.

In France, neonatal SCD screening is restricted to newborns whose parents come from a defined list of at-risk countries with regard to the SCD trait (https://www.has-sante.fr/plugins/ModuleXitiKLEE/types/FileDocument/doXiti.jsp?id=c_1724722). This screening is recommended if (i) one or both parents come from an at-risk region; (ii) there is a family history of SCD; or (iii) there is any doubt concerning these criteria. For this reason (and in the absence of an index case), most adults with the HbAS/HbAC trait in France become aware of their status following neonatal screening of their child. In such an event, the parents receive a letter comprising a standard information sheet and an invitation to make an appointment for screening. In the Paris Ile-de-France region, the data for 2015 show that 238 children were diagnosed with major SCD and 5,632 newborns were identified as HbAS or HbAC carriers. In 2006, the Paris Federation for the Screening and Prevention of Child Handicap (*Fédération Parisienne pour le Dépistage et la Prévention du Handicap chez l'Enfant*, FPDPHE) initiated a new programme in which all parents of HbAS/HbAC screened newborns were contacted by post. The programme's objectives were to (i) give the parents the newborn's screening results, (ii) provide information on SCD, and (iii) recommend an appointment with a specialist for parental screening and genetic counseling.

The limitations of this information programme (2008–2011) were recently described by Lainé et al. (2). The researchers reported that around 10% of couples did not receive the letter (due to an incorrect address), and that 80% had received the letter but had not taken any further action. Overall, only 8% of the couples followed the recommendations outlined in the letter.

The primary objective of the present study was to assess the putative added value (in terms of a higher screening rate for at-risk parents) of following up the “carrier status” letter with a phone call or a text message, relative to a letter alone.

## Methods

We performed a single-center, randomized, open study of three ways of providing information to 300 couples after their child had been identified as a sickle cell carrier. This study was sponsored by the Assistance Publique-Hôpitaux de Paris (AP-HP) and approved by the local independent ethics committee (*CPP Ile-de-France II*, Paris, France; reference: 07/12/2015). The requirement for written informed consent was waived by the ethics committee because neither supplementary sampling nor supplementary blood tests were performed for this study. This non-interventional research study consisted in contacting 300 couples of parents after their child had been identified as a SCD carrier in the French national newborn screening program (in order to increase their risk knowledge) otherwise than through a conventional information letter.

The parents' demographic data (including a full postal address and phone numbers) were collected at the maternity ward immediately after birth, when the neonatal screening request and parental consent are collected. All the demographic data and the card test were sent to the FPDPHE. In line with French legislation, the FPDPHE must send an information letter to the parents if the child's screening test is positive.

Thus, in line with the FPDPHE's standard procedure, 300 letters were sent to couples of parents of newborns identified as SCD carriers (i.e., heterozygotes for abnormal hemoglobin).

Most of these children carried the HbAS or HbAC trait, although carriers of other abnormal haemoglobins (e.g., HbAE, HbAD, and HbAX, where X corresponds to an unidentified hemoglobin) were also included. The letter informed the parents of their child's carrier status, offered a screening appointment, and provided information about the present study. The sample size of 300 based on a putative 20% relative increase in the screening rate for parents having received a follow-up phone call or text message.

The 300 couples were randomized as follows: 100 were sent a letter only (arm A), 100 were sent a letter that was followed up by a phone call (arm B), and 100 were sent a letter that was followed up by a three text messages at 5-day intervals (arm C). The content of the letter, the explanations provided by phone, and the text messages are given as Supplementary Data. The parents were phone or messaged 2 weeks after the letter was sent. This time interval enabled us to quantify the proportion of patients who spontaneously contacted or phoned Necker Children's Hospital in response to the information letter alone (i.e., the letter's efficacy).

One or both parents were phoned or messaged, depending on which phone numbers they had given to the maternity unit.

We checked that all the parents who answered the phone call understood the explanations provided and the medical terms used by the genetic counselor well enough. Parents were invited to make an appointment for a consultation with a hematologist and a genetic counselor and for screening at Necker Children's Hospital (Paris, France). This invitation did not prevent parents from requesting a consultation and screening at another healthcare center (one closer to their home, for example). The cost of these consultations (regardless of the provider) was reimbursed by the French National Health Insurance systems. Parents having been screened prior to the neonatal screening and parents not wishing to participate in the study were excluded from our analysis. This information was collected through a phone call at the end of the study (i.e., ~120 days after the letter had been sent).

The primary endpoint was the number of parents in each arm screened (regardless of the screening center) in the 120 days after the letter had been sent. The secondary endpoints were as follows: (i) the number of parents having spontaneously contacted the screening center at Necker Children's Hospital by phone in the 2 weeks after the letter had been sent, (ii) the number of consultations by these parents at Necker Children's Hospital, (iii) the number of parents screened at Necker Children's Hospital, (iv) the number of consultations related to the newborn's heterozygous status at other healthcare centers, (v) the number of parents screened at other healthcare centers and (vi) the number of parents screened. This information was gathered by phoning parents (regardless of the study arm) who had not contacted the study site in the 120 days after the letter had been sent. Parents who had undergone screening prior to the neonatal screening were excluded from the analysis.

The study was performed between 2015 and 2017 by staff in the Biotherapy Department and the Genetics Department at Necker Children's Hospital (Paris, France), the FPDPHE, and the Necker-Cochin-Tarnier Clinical Research Unit (Paris, France), as part of a well-structured network.

### Statistics

The required sample size was calculated by assuming that the screening rate would be 8% in arm A. With a two-sided alpha risk of 0.05, a power of 90%, and a prior screening rate of 20%, we calculated that 300 couples would have to be randomized to detect an absolute difference of 20 percentage points in the screening rate between arm B or arm C on one hand and arm A on the other.

The data are presented here as the number (%) for qualitative variables and as the median [interquartile range (IQR)] (range) for quantitative variables. Values of quantitative variables were compared using non-parametric Wilcoxon tests, while proportions of qualitative variables were compared using chi-squared tests. Unless otherwise stated, the threshold for statistical significance was set to *p* < 0.05. All tests were two-sided.

## Results

### Characteristics of the Study Population

**Figure 1:**
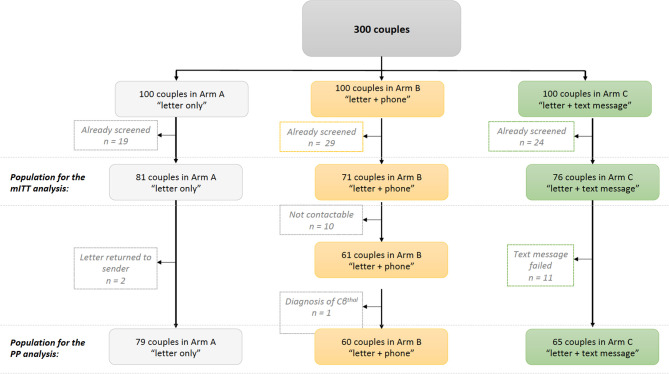
Study flow chart. mITT: modified intention-to-treat; PP: Per protocol.

Of the 300 couples, 72 had been screened before the start of the study and so were excluded from our analyses (19 in arm A, 29 in arm B, and 24 in arm C; *p* = 0.25 for the inter-arm difference). These parents had been screened during pregnancy, as part of a family survey or because they already knew that they were at risk of transmitting SCD. Thus, 81 couples in arm A, 71 couples in arm B, and 76 couples in arm C constituted the study population for our modified intention-to-treat (mITT) analysis (Figure 1). The three arms did not differ significantly with regard to the newborn's age when the letter was sent (mean age: 33 days; *p* = 0.90), the distribution of the newborns' hemoglobin profiles (*p* = 0.41), the grade of the maternity unit where the child was born (*p* = 0.78), and the parents' county of residence in the Paris Ile-de-France region (*p* = 0.93) (Table 1).

**Table 1:** Characteristics of the study population.

	**Arm A** **(*n* = 81)**	**Arm B** **(*n* = 71)**	**Arm C** **(*n* = 76)**	***p*-value**
	**Letter only**	**Letter + phone** **follow-up**	**Letter + text** **message follow-up**	
**Child's age when the letter was sent (in days)**	33 [29–36] (25–44)	33 [29–37] (26–44)	33 [29–36] (25–43)	0.9
**Child's hemoglobin profile**				0.41
AS	55 (67.9%)	40 (56.3%)	53 (69.7%)	
AC	16 (19.8%)	20 (28.2%)	15 (19.7%)	
AD	3 (3.7%)	2 (2.8%)	0 (0%)	
AE	2 (2.5%)	5 (7%)	2 (2.6%)	
AX	5 (6.2%)	4 (5.6%)	6 (7.9%)	
**Parents' county of residence**				0.93
Paris	8 (9.9%)	10 (14.1%)	8 (10.5%)	
Seine-et-Marne	8 (9.9%)	4 (5.6%)	3 (3.9%)	
Yvelines	7 (8.6%)	6 (8.5%)	6 (7.9%)	
Essonne	11 (13.6%)	7 (9.9%)	11 (14.5%)	
Hauts-de-Seine	8 (9.9%)	5 (7%)	9 (11.8%)	
Seine-Saint-Denis	9 (11.1%)	12 (16.9%)	14 (18.4%)	
Val de Marne	15 (18.5%)	14 (19.7%)	9 (11.8%)	
Val d'Oise	15 (18.5%)	12 (16.9%)	15 (19.7%)	
Eure-et-Loir	0 (0%)	1 (1.4%)	1 (1.3%)	
**Maternity unit grade**				0.78
1	10 (12.3%)	9 (12.7%)	12 (15.8%)	
2	44 (54.3%)	38 (53.5%)	34 (44.7%)	
3	27 (33.3%)	24 (33.8%)	30 (39.5%)	

*Grade 1: obstetric unit*.

*Grade 2: obstetric unit + neonatal care unit*.

*Grade 3: obstetric unit + neonatal care unit + neonatal intensive care unit + neonatal resuscitation facility*.

*Data are presented as the median [IQR] (range) or n (%)*.

In a per-protocol (PP) analysis, we excluded two couples in arm A whose letters had been returned to the sender, 10 couples in arm B who could not be contacted, and 11 couples in arm C who did not receive the text messages (i.e., text messages returned to the sender). Another couple was excluded from arm B following the discovery of an erroneous diagnosis (a child with HbCβ^thal^ and not HbAC). Hence, the PP population comprised 79 couples in arm A, 60 in arm B, and 65 in arm C (Figure 1).

Lastly, 20 couples in arms A, 19 in arm B, and 27 in arm C could not be contacted 120 days after the letter had been sent; hence, we were unable to gather any data on the outcome for these parents.

### Analysis of the Primary Endpoint

The mITT analysis of the primary endpoint revealed a parental screening rate of 17% in arm A, 35% in arm B (*p* = 0.019 vs. arm A), and 30% in arm C (*p* = 0.08 vs. arm A). The PP analysis of the primary endpoint gave similar results, with a screening rate of 18% in arm A, 40% in arm B (*p* = 0.006 vs. arm A), and 35% in arm C (*p* = 0.02 vs. arm A). The difference between the screening rates in arms B and C was not significant in the mITT or PP analysis (*p* > 0.05). Hence, follow-up with a phone call or a text message was associated with a significantly higher screening rate among parents of newborns screened as HbAS/HbAC, relative to the standard procedure (a letter only) (Table 2).

**Table 2:** Screening rates for parents of sickle cell carriers.

	**Arm A**	**Arm B**	**Arms C**	***p*****-value**	
**Analysis of the primary endpoint**				**B vs. A**	**C vs. A**
Modified intention-to-treat population	*n* = 81	*n* = 71	*n* = 76		
Number of newly screened parents	14 (17%)	25 (35%)	23 (30%)	0.019	0.08
Per protocol population	*n* = 79	*n* = 60	*n* = 65		
Number of newly screened parents	14 (18%)	24 (40%)	23 (35%)	0.006	0.02

### Analysis of the Secondary Endpoints

**Figure 2:**
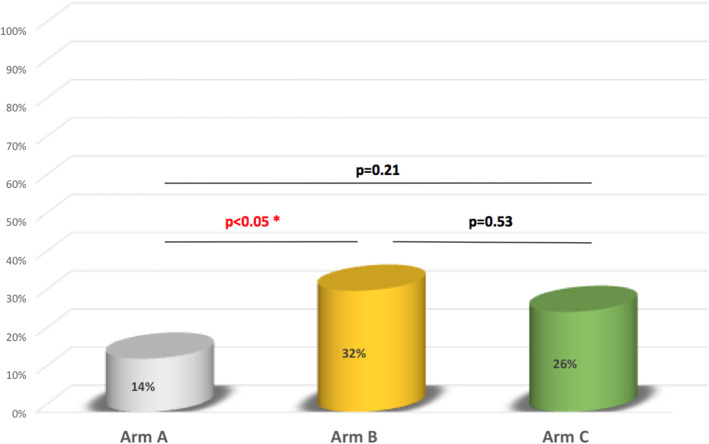
couple screening rates (i.e. both parents).

In the 2 weeks after the letter had been sent, the spontaneous response rate was 6.3% in arm A, 5% in arm B, and 4.6% in arm C. Ten of the 27 couples in arm A (37%), 7 of the 11 in arm B (63.3%), and 6 of the 11 in arm C (54.5%) reported that they had been screened in a healthcare center other than Necker Children's Hospital. All the couples who consulted at Necker Children's Hospital (all four in arm A, all 17 in arm B, and all 17 in arm C) were subsequently screened. At Necker Children's Hospital and other centers either one or both of the parents were screened (Table 3). The proportion of couples in which both parents were screened was 14% in arm A, 32% in arm B, and 26% in arm C. The difference in this proportion between arms B and C was not statistically significant (*p* = 0.53) (Figure 2).

**Table 3:** Secondary endpoints.

	**Arm A** ***n* = 79**	**Arm B** ***n* = 60**	**Arm C** ***n* = 65**	***p*-value**
Number of calls received spontaneously	5 (6.3%)	3 (5%)	3 (4.6%)	0.93
Number of consultations at NCH	4 (5.1%)	17 (28.3%)	17 (26.2%)	0.00013
- number of parents screened at NCH	4 (100%)	17 (100%)	17 (100%)	–
Number of consultations outside NCH	27 (34.2%)	11 (18.3%)	11 (16.9%)	0.03
- number of parents screened in consultations outside NCH	10 (37%)	7 (63.6%)	6 (54.5%)	0.28

*NCH, Necker Children's Hospital*.

### A Longitudinal Analysis of the Process Leading to Screening of Parents of HbAS/HbAC Newborns

**Figure 3:**
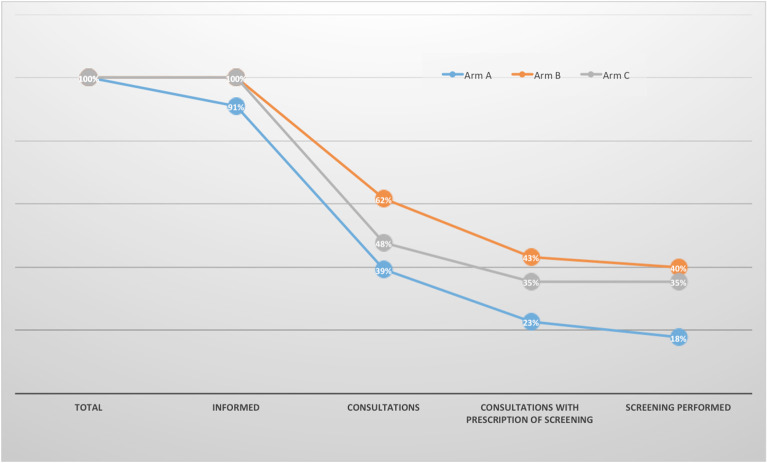
Reasons for non-screening. Informed: couples having received the information that their newborn carried the HbAS/HbAC trait by one of the three methods (letter only, letter + phone or Letter + text message).

We identified four successive steps in the screening process: the provision of information to the parents, the parents' request for a consultation with a specialist physician, the referral for screening at the end of the consultation, and the screening itself. We then calculated the proportion of parents assessed at each step. All the parents in arms B and C were successfully informed of their child's carrier status for sickle cell disease, vs. 91% in arm A (i.e., seven parents in arm A claimed they had not received the letter). It is noteworthy that 45% of the parents having received the letter claimed that the latter was not sufficiently clear on why they should seek to be screened. The consultation attendance rate was 39% in arm A, 62% in arm B, and 48% in arm C. The rate of referral (i.e., an appointment) for screening was 23% in arm A, 43% in arm B, and 35% in arm C. Lastly, the actual screening rate was 18% in arm A, 40% in arm B, and 35% in arm C (Figure 3).

## Discussion

For the parents of a child carrying the SCD trait, genetic counseling is a crucial step in the prevention of this severe, life-threatening disease and in the provision of information on health risks. An earlier single-center study reported that only 8% of parents (429 out of 5,379) of an HbAS/HbAC carrier responded to a letter informing them of the neonatal screening results. However, the latter single-center study was limited by the fact that it did not take account of consultations performed in other healthcare centers (2). These observations prompted us to consider ways of improving the screening rate.

An mITT analysis of our present results showed that telephone and text message follow-ups were associated with a significantly higher screening rate among parents of children screened as HbAS/HbAC, relative to the standard “letter only” procedure. A PP analysis confirmed the significantly higher screening rate in arm B (telephone follow-up, 40%) and arm C (text message follow-up, 35%) than in arm A (letter only, 18%). In contrast, the difference between arms B and C was not significant.

It should be noted that the study's methodology prevented us from performing a standard (non-modified) ITT analysis; we did not know whether the parents had been screened prior to randomization. So as not to bias the proportion of newly screened parents in a standard ITT analysis, we decided to exclude these parents and therefore performed an mITT analysis.

When considering only consultations performed outside Necker Children's Hospital, we observed a trend toward a higher screening rate in arms B and C than in arm A (63.6, 54.5, and 37%, respectively). However, the small sample size meant that this difference was not statistically significant. Furthermore, it appeared that a referral for hemoglobin analysis prompted the parents to undergo screening. When considering parents who had been referred for screening, the proportion of fulfilled prescriptions was 95% in arm A, 97% in arm B, and 100% in arm C.

In order to identify couples at risk of transmitting major sickle cell syndrome, both parents have to be screened. At-risk couples are offered a consultation with a genetic councilor and a specialist physician, in order to inform them of the available options: treatment of a child with sickle cell disease in a reference center, invasive prenatal diagnosis, preimplantation genetic diagnosis, and the storage of umbilical cord blood for subsequent autologous gene therapy (if and when this procedure becomes widely available).

In the present study, the proportion of couples for which both parents were screened was higher in arm B (by 32 percentage points; *p* < 0.05) and in arm C (although not significantly) than in arm A. The added value of telephone follow-up in a screening programme has been reported in the literature (3–7).

We identified two couples (one in arm B and one in arm C) at risk of having a child with major sickle cell syndrome. Although couple screening is the gold standard for accurately estimating the risk of subsequently having a child with major sickle cell syndrome, the high frequency of single-parent families in the present study prompted us to measure the number of parents screened as our primary endpoint.

According to the phone call made 120 days or more after the letter had been sent, 25% of the parents in the whole study population knew their hemoglobin status prior to receipt of the letter. These parents had been screened during pregnancy or during a family survey or were otherwise aware that they were at risk of transmitting sick cell disease. Furthermore, 34.2% of the parents in arm C had consulted a healthcare professional but had not been screened.

We therefore focused on the reasons for non-screening among parents of sickle cell carriers. Some non-screened parents had not received the letter or had not fully understood its potential relevance with regard to health. Other parents had shown the letter to a general practitioner or a pediatrician (regardless of whether they had understood it or not) but this was not always followed by a prescription for hemoglobin screening.

Furthermore, the phone interview 120 days or more after the letter had been sent demonstrated that in many cases, parents were reassured about their child's state of health but had not referred for parental screening; 16% of the parents in arm A, 19% in arm B, and 13% in arm C had consulted a physician but had not been referred for hemoglobin screening. The importance of training physicians to manage this situation has been reported previously (8).

Neonatal screening programmes are designed to identify newborns with sickle cell disease (https://www.has-sante.fr/plugins/ModuleXitiKLEE/types/FileDocument/doXiti.jsp?id=c_1724722) (9). However, they also identify healthy children who carry the HbAS/HbAC trait. Since the introduction of neonatal sickle cell disease screening programmes, the rationale for informing parents of their child's HbAS/HbAC carrier status has been subject to ethical and sociological debate (5).

In conclusion, we consider that a follow-up phone call or text message provided useful information to at-risk parents and guided them in their future parental choices. Screening rates would probably be increased further by (i) effective training of all the healthcare professionals involved in this process, and (ii) setting up a telephone hotline for parents who have been informed of their child's carrier status.

## Data Availability Statement

All datasets generated/analyzed for this study are included in the article/Supplementary Material.

## Ethics Statement

This study received ethical approval from the local independent Ethics Committee (CPP Ile-de-France II, Paris, France; reference: 07/12/2015). The requirement for written informed consent was waived by the Ethics Committee because neither supplementary sampling nor supplementary blood tests were made for this study.

## Author Contributions

RG and MC supervised and designed the project. CR and AS performed data analysis and interpretation. RG, MC, CR, and AS wrote the manuscript. NB, VG, MP, SB, AN, EG, J-MT, and AM were physician in charge of the patients.

## Conflict of Interest

The authors declare that the research was conducted in the absence of any commercial or financial relationships that could be construed as a potential conflict of interest.
