# Supplementary material for: An Evaluation of Three Ways of Communicating Carrier Status Results to the Parents of Children in a Neonatal Sickle Cell Screening Programme
Source: Front Pediatr. 2020 Jun 19;8:300. doi: 10.3389/fped.2020.00300 (PMC7318296; doi:10.3389/fped.2020.00300)
Supplement: Supplementary file 1 [file Data_Sheet_1.PDF]

The impact of different information channels (a letter, a phone call, and a text message) on parental screening after a neonate has been identified as a heterozygous sickle cell disease carrier (the "DrepaSMS" study).

STUDY INFORMATION SHEET

Madam, Sir,

Professors Marina Cavazzana and Arnold Munnich (respectively heads of the Department of Biotherapy and the Department of Genetics at Necker Children's Hospital) have invited you to participate in a study of the parents of children having been identified as healthy sickle cell carriers ("AS heterozygous").
It is important to read this note carefully before making your decision; do not hesitate to ask them *[Professors Cavazzana and Munnich]* for further explanations.

1) What is the study's objective?

This study deals with improving screening and information provision for the parents of newborn babies identified as healthy sickle cell carriers. It will help to identify couples at risk of having a child with full sickle cell disease in the future. In order to address the study's objective, we plan to include 300 couples (the mother and the father) with a heterozygous child. The study will be carried out at Necker Children's Hospital.

2) What does the study involve?

This study consists in randomly forming 3 groups of parents of new-borns screened as healthy AS sickle cell carriers:

1) A control group: the parents in this group will receive a letter inviting them to make an appointment for a genetic counselling consultation so that they can receive information and be screened. The letter is attached to this study information sheet.
2) A "phone call" group: the parents in this group will receive the same letter as the control group and then a follow-up phone call two weeks later.
3) A "text message" group: the parents of this group will receive the same letter as the control group and then 3 follow-up text messages two weeks later.

The objective of the research is to compare the 3 groups of parents with regard to the number of genetic counselling consultations, in order to evaluate the impact of a follow-up telephone call or text message after receipt of the letter.

3) What is the study's timeline?

The study will last for a year, and your participation will last for a month (the maximum time interval between receipt of the letter and the genetic counselling consultation).

4) What are the benefits and constraints of participation in the study?

This study does not feature any constraints. Its goals are to promote screening, to find out the haemoglobin status of your couple, and to provide you with genetic counselling. During the consultation, the genetic counsellor will explain your options if you are both healthy AS sickle cell disease carriers:

- Care provision for a sick child, with the possibility of storing some umbilical cord blood for autologous gene therapy in the future.
- Prenatal genetic diagnosis.

- Preimplantation genetic diagnosis.

5) If I participate, what will happen to the personal data collected in the study?

As part of the biomedical research in which Professors Cavazzana and Munnich (under the sponsorship of our institution, Assistance Publique Hôpitaux de Paris (AP-HP)) are inviting you to participate, your personal data will be processed so that the study results can be analyzed.
These data will be identified by a code number and your initials. The data might also be sent (under confidential conditions) to the French healthcare authorities.
If you stop participating in the study but do not withdraw your consent, the data collected before you have stopped will be processed – unless you object to this.

6) What is the study's legal framework?

The AP-HP has complied with all the provisions of the French legislation on biomedical research (the Huriet Act 88-1138 dated December 20th, 1988, as modified by the Public Health Act 2004-806 dated August 9th, 2004).

The AP-HP obtained approval for the study from the local independent ethics committee (*Comité de Protection des Personnes Ile de France II)* on November 9th, 2015.

7) What are my rights?

You are entirely free to participate or not in this research. You can decide to stop participating in the study at any time.

In compliance with the provision of the French legislation on data processing, you have the right to access and rectify your personal data. You also have the right to object to the transmission of the confidential data to be processed as part of this research. To exercise these rights, contact Professors Cavazzana and Munnich.
Your medical records will remain confidential and will only be consulted by the physician managing your treatment, by the healthcare authorities and by study personnel duly mandated by the AP-HP and who have a professional duty of confidentiality.

At the end of the study and after the study data have been analyzed, you will be informed about the study's overall results by the physician who is monitoring you as part of this research.

For any additional information, contact:

Professor Marina Cavazzana – Head of the Department of Biotherapy - Necker Children's Hospital, 149 rue de Sèvres, 75743 Paris cedex 15, France – Tel.: +33-144-495-068

or

Professor Arnold Munnich - Head of the Department of Genetics - Necker Children's Hospital,149, rue de Sèvres, 75743 Paris cedex 15, France - Tel.: +33-144-495-151
