# Supplementary material for: An Evaluation of Three Ways of Communicating Carrier Status Results to the Parents of Children in a Neonatal Sickle Cell Screening Programme
Source: Front Pediatr. 2020 Jun 19;8:300. doi: 10.3389/fped.2020.00300 (PMC7318296; doi:10.3389/fped.2020.00300)
Supplement: Supplementary file 2 [file Data_Sheet_2.PDF]

Fédération Parisienne pour le Dépistage et la Prévention des Handicaps de l'Enfant

Necker ENFANTS MALADES HÔPITAL UNIVERSITAIRE

FPDPHE file number:

Recipient (name, first name; address)

Paris, (Date)

Madam, Sir,

Your child [name] born on [date] at the [maternity unit name] maternity unit has been screened for sickle cell disease.

The screening result shows that he/she is **not ill but is a healthy carrier**.
This result means that we now wish to screen you (the child's two parents) for sickle cell disease status.

To do this, you can either:

- **Show** this letter to a healthcare professional of your choice: a paediatrician at the maternity unit or paediatric clinic, or the physician who looks after your child (a paediatrician or general practitioner).
- **Attend** the Sickle Cell Disease Information and Screening Centre **without making an appointment** (**completely free**).
- **Make an appointment for a genetic counselling consultation** by calling the phone number below:

Genetic Counselling Consultation
Necker Children's Hospital, 149 rue de Sevres
F-75015 Paris, France
Tel.: +33-142-754-799

Do not hesitate to contact us for more information.

Yours faithfully,

Professor Robert Girot
Head Physician for genetic counselling consultations at Necker Children's Hospital

Christelle Remus
Genetic Counsellor
Necker Children's Hospital
