# Supplementary material for: An Evaluation of Three Ways of Communicating Carrier Status Results to the Parents of Children in a Neonatal Sickle Cell Screening Programme
Source: Front Pediatr. 2020 Jun 19;8:300. doi: 10.3389/fped.2020.00300 (PMC7318296; doi:10.3389/fped.2020.00300)
Supplement: Supplementary file 3 [file Data_Sheet_3.PDF]

Supplementary Data : the template of the SMS sent to the parents

Hello,
Your baby is a healthy sickle cell disease carrier. We recommend that as parents, you should make an appointment by calling 01 42 75 47 99.
